# Transcriptome signature for dietary fructose-specific changes in rat renal cortex: A quantitative approach to physiological relevance

**DOI:** 10.1371/journal.pone.0201293

**Published:** 2018-08-01

**Authors:** Agustin Gonzalez-Vicente, Jeffrey L. Garvin, Ulrich Hopfer

**Affiliations:** Department of Physiology & Biophysics, Case Western Reserve University, Cleveland, OH, United States of America; George Washington University School of Medicine and Health Sciences, UNITED STATES

## Abstract

Fructose consumption causes metabolic diseases and renal injury primarily in the renal cortex where fructose is metabolized. Analyzing gene differential expression induced by dietary manipulation is challenging. The effects may depend on the base diet and primary changes likely induce secondary or higher order changes that are difficult to capture by conventional univariate transcriptome analyses. We hypothesized that dietary fructose induces a genetic program in the kidney cortex that favors lipogenesis and gluconeogenesis. To test this, we analyzed renal cortical transcriptomes of rats on normal- and high-salt base diets supplemented with fructose. Both sets of data were analyzed using the Characteristic Direction method to yield fructose-induced gene vectors of associated differential expression values. A fructose-specific “signature” of 139 genes differentially expressed was extracted from the 2 diet vectors by a new algorithm that takes into account a gene’s rank and standard deviation of its differential expression value. Of these genes, 97 were annotated and the top 34 accounted for 80% of the signal in the annotated signature. The genes were predominantly proximal tubule–specific, coding for metabolic enzymes or transporters. Cosine similarity of signature genes in the two fructose-induced vectors was >0.78. These 139 genes of the fructose signature contributed 27% and 38% of total differential expression on normal- and high- salt diet, respectively. Principal Component Analysis showed that the individual animals could be grouped according to diet. The fructose signature contained a greater enrichment of Gene Ontology processes related to nutrition and metabolism of fructose than two univariate analysis methods. The major feature of the fructose signature is a change in metabolic programs of the renal proximal tubule consistent with gluconeogenesis and de-novo lipogenesis. This new “signature” constitutes a new metric to bridge the gap between physiological phenomena and differential expression profile.

## Introduction

Elevated consumption of fructose has been associated with metabolic disorders and salt-sensitive hypertension [1]. However, the connection between fructose metabolism and the pathogenesis of fructose consumption-associated diseases remains unresolved and controversial [2]. An interesting hypothesis is that the abundance of fructose in fruits occurring at the end of summer or wet-season preludes times of food scarcity, and may have been selected for by evolution as a trigger for fat accumulation; the “fat switch” [3, 4]. Such altered metabolism and appetite, seen, for example in hibernation, are genetically programmed mechanisms. Although there may have been an adaptive advantage to the “fat switch” in evolutionary terms, in the present environment of food abundance even moderate amounts of fructose may lead to pathological conditions including renal failure, hypertension, and cardiovascular disease [5, 6].

The renal cortex can contribute significantly to plasma glucose and lipids depending on metabolic state. The proximal tubule of the nephron is the only renal tissue that expresses fructokinase (ketohexokinase) [7, 8] and catabolizes fructose [9, 10]. Cells of this segment can use the carbon backbone of fructose to synthesize both glucose and lipids, and in the Krebs cycle to generate ATP. We have previously shown that dietary fructose (20% fructose beverage for 7 days) enhances the ability of angiotensin II to stimulate Na reabsorption in this segment [11], a process dependent on oxidative phosphorylation. However, it is unknown whether dietary fructose initiates a genetic program in the renal cortex that favors an altered metabolic state and lipogenesis, as in the liver [12].

Transcriptome signatures are proving useful in cancer, immunology and other fields [13–15], but have yet to be applied in detail to nutritional problems. This absence is explained, at least partially, by the greater complexity of metabolic changes in response to dietary interventions. There are several problems that need to be addressed to obtain informative data of differential expression (DE) of genes when assessing the effect of dietary manipulations [16]. First, the effects of adding or changing a nutrient to a base diet will depend on the composition of the base diet and amount and length of supplementation. Second, metabolic states are influenced by a number of poorly or uncontrollable variables, such as the gut microbiome or circadian rhythm, that increase the variance of baseline gene expression. Third, changes in diet would be expected to lead to primary metabolic changes, which then trigger higher order DE of other genes. The serial responses could result in a potentially large number of small changes in gene expression that are difficult to identify, but, nevertheless, are relevant to the physiology. Such changes are not likely captured by univariate analyses. Furthermore, no consensus currently exists on the criteria by which to identify the complete set of differentially expressed genes.

To overcome many of the problems associated with such studies we: 1) used a two-by-two design in which the effects of fructose were examined on two base diets; 2) converted expression data to gene vectors with associated DE values with the multivariate Characteristic Direction (CD) method [17]; and 3) developed a new algorithm that combines the results from two base diets and identifies a cut-off for differentially expressed genes called “rank/deviation”. The primary focus of this report is on evaluation of the effectiveness of this approach over currently used methods in identifying the gene programs expressed in response to fructose.

Here we report for the first time the effects of dietary fructose on DE of genes in the renal cortex. Most of the highest ranked DE genes relate to enhanced gluconeogenesis and lipogenesis. These processes are known to occur in the proximal tubule under some metabolic conditions, but the extent of fructose control of these processes has not been investigated. Principal Component Analysis and Cosine Similarity confirmed effectiveness of our approach in identifying DE genes. Advantages of our methods were demonstrated by comparing results to those from two conventional univariate analyses: ANOVA and Bayesian Analysis of Variance for Microarrays (BAM) [18–20]. Most importantly, the CD method allows identification of gene programs, whereby the relative expression changes of genes in response to a stimulus constitute a new quantitative metric to bridge the gap between physiological phenomena and differential expression signatures.

## Materials and methods

### Animal model and sample collection

**Fig 1:**
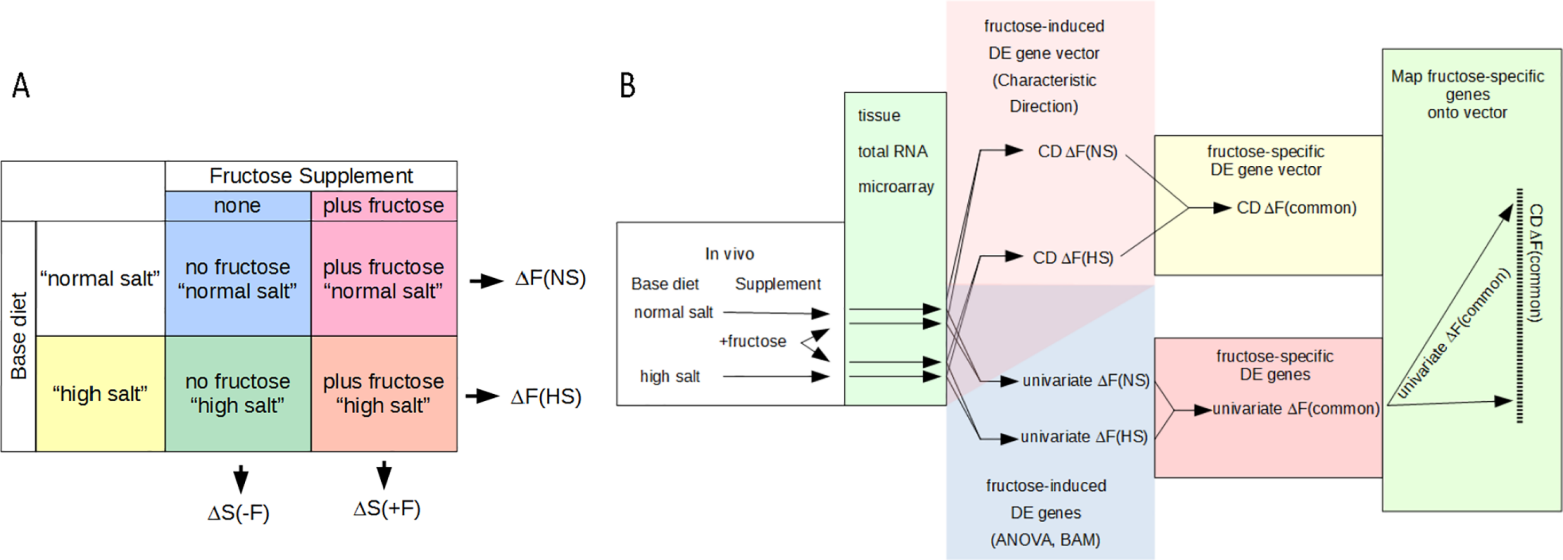
Outline of experimental design and analysis. A) 2 x 2 experimental design of the animal food with 2 different base diets supplemented with 20% fructose in the drinking water. The 2 x 2 design allows analysis of fructose-induced DE effects on both normal- and high-salt base diets. These results are designated as ΔF(NS) and ΔF(HS), respectively. It also allows an analysis of expression changes due to consumption of different base diets, both in the absence or presence of supplemental fructose. The results from this type of analysis are designated as ΔS(-F) and ΔS(+F), respectively, because the normal-salt diet is used as control. B) Transcriptome analysis scheme to extract a fructose-specific DE vector (signature) by the multivariate CD method (upper analysis flow) and generate gene lists by the univariate methods of ANOVA and BAM (lower analysis flow). The multivariate and univariate methods result in different types of primary data (middle column): CD generates a vector with a DE value (*v*_*i*_) for each gene, which is normalized so that $\sum_{i=1}^{n}{\left(v_{i}\right)}^{2}\equiv 1$. The univariate methods of ANOVA and BAM generate lists of DE genes, depending on the cut-off criteria (e.g., p ≤ 0.05 for ANOVA). To find fructose-specific DE that is common to both base diets, the information from ΔF(NS) and ΔF(HS) is combined. With CD, a gene vector ΔF(common) is created with DE values as geometric means of those of ΔF(NS) and ΔF(HS). The genes with the top most DE values, low enough variance, and similar effects on the two base diets are extracted to yield CD ΔF(signature) as detailed in Fig 2. In the univariate methods, genes with fructose-specific DE are identified by applying the significance criteria simultaneously to both ΔF(NS) and ΔF(HS) and omitting genes with changes in opposite direction. Those fructose-specific genes identified by ANOVA and BAM that are part of the fructose-specific CD ΔF(signature) are then mapped onto this signature, i.e., located on the vector CD ΔF(signature) (last column).

Fig 1 provides an outline of experimental design and analysis. To overcome the problem of a dietary supplement-base diet interaction, we analyzed differential gene expression in response to fructose supplementation using rats on two different base diets (Fig 1A) producing four treatment groups of 4 animals each. The normal-salt base diet contained 0.60% NaCl (Prolab Isopro RMH 3000, 22.5% protein, 5.5% fat, 51.6% carbohydrates and an energy content of 4.18 kcal/g) while the high-salt diet contained 4% NaCl (Harlan TD.92034, 19.4% protein, 5.2% fat and 47.5% carbohydrates, providing 3.1 kcal/g). Two groups received 20% fructose in the drinking water for 7 days to catch early effects of fructose before onset of the known chronic pathologies [12]. Base diets and fructose delivery protocols were based on a well-characterized model [5, 21]. The study was approved by the Institutional Animal Care and Use Committee of Case Western Reserve University. All experiments were conducted in accordance with the National Institutes of Health Guidelines for the Care and Use of Laboratory Animals.

Experimental animals were male Sprague-Dawley rats (Charles River Breeding Laboratories, Wilmington, MA) weighing between 101 and 125 g. They were allowed 5 days for acclimation to the facility and housed in pairs under normal rat housing conditions with a 12-hour light cycle and access to food and fluids *ad libitum*. They were randomly assigned to one of four groups.

Because of the previous characterization of the animal model, only weight was monitored in this study as surrogate for the beginning of fructose-induced obesity. This parameter was normal for all groups and showed no difference between groups (S8 Table).

After 7 days of the treatment, rats were anesthetized with ketamine (100 mg/kg bw IP) and xylazine (20 mg/kg bw IP), and given 2 IU heparin (IP). Surgical instruments and working space were decontaminated with RNase Away Reagent (Life Technologies, #10328–011). The abdominal cavity was opened and the kidneys cooled-down by pouring cold saline on them. Then, the right kidney was excised, rapidly submerged in a large volume of cold saline, and transferred to a trans-illuminated acrylic plate at 4°C. The tissue sample was a ~1 mm-thick slice of superficial cortex from a sagittal cut. The sample was immediately transferred to a tube containing 1 ml RNAlater (Qiagen; #76104) and kept at -20° C until extraction. Total RNA was extracted using miRNeasy Mini Kit (Qiagen; #217004) according to the manufacture’s recommendations. Quality control was assessed by measuring the 260/280 absorbance ratio. All analyzed samples had 260/280 ratios > 1.8.

### Microarray data

Microarray data were generated from the total RNA by the Gene Expression and Genotyping Facility (GEGF.net) of the Case Comprehensive Cancer Center according to the Affymetrix WT Plus protocol and gene chip *RaGene*-2_0-st. This chip uses 214,954 probes in 30,472 TranscriptClusterIDs, 62.2% of which are annotated. The data are available in the NIH GEO database under GSE103110 (https://www.ncbi.nlm.nih.gov/geo/query/acc.cgi?acc=GSE103110).

Analyses of the Affymetrix image files were carried out on a desktop computer with windows 8.1 as operating system. The image files were converted to expression data and normalized by Expression Console build 1.4.1.46 using the *RaGene*-2_0-st data file and the robust multi-array average method. Other parameter settings included use of quartile for normalization and median polish for summarization. Further analyses to extract differentially expressed genes were carried out with expression data at the TranscriptClusterID (gene) level and conversion to gene symbols was made only in the final steps using RaGene-2_0-st-v1.na36.rn5.transcript.csv.

### Microarray data analysis with the Characteristic Direction (CD) method

In an attempt to overcome the problem of identifying important genes in spite of high variance of baseline expression, we used the multivariate CD method for analysis [17] implemented on R [22]. The CD method calculates a vector “*V”* of a differential expression value (DE value) for each TranscriptClusterID between groups “a” and “b” as follows:

$$V=\sum^{-1}\times \left(\mu_{a}-\mu_{b}\right)$$ (1)

Whereby Ʃ is the covariance matrix of the data, and μ_a_ and μ_b_ are the vectors of the means of the expression values of each TranscriptClusterID from the experimental groups “a” and “b”, respectively (Fig 1A). Four gene vectors with associated DE values were generated: 1) effect of fructose on a normal-salt diet: CD ΔF(NS); 2) effect of fructose on a high-salt diet: CD ΔF(HS); 3) effect of the high- versus the normal-salt diet in the absence of fructose: CD ΔS(-F); and 4) in the presence of fructose: CD ΔS(+F). Then each of the 4 vectors (with elements DE value_*i*_ = *v*_*i*_) was normalized as follows:

$$\sum_{i=1}^{n}{\left(v_{i}\right)}^{2}\equiv 1$$ (2)

with “*i*” ranging from 1 to *n*, where “n” represents the number of genes.

The normalized vector values quantitatively reflect the relative contribution to differential expression of all genes. To easily extract the most important genes, vectors are sorted in descending order of the absolute of the squared *v*_i_ value and genes are indexed so that “*i*” indicates the positional rank after sorting.

### Identifying the common fructose signature

Fig 1B outlines our strategy for identifying a common signature of DE for the fructose effect on the two base diets. To combine the information from the CD ΔF(NS) and CD ΔF(HS) vectors, a secondary vector CD ΔF(common) with common values “*vc*” was generated by gene-wise calculating the geometric mean of the values (v_i_) of ΔF(NS) and ΔF(HS), whereby the geometric mean of two numbers *v1*_*i*_ and *v2*_*i*_ is defined as follows:

1. if *v1*_*i*_ and *v2*_*i*_ are greater than zero, $vc_{i}=+\sqrt{v1_{i}*v2_{i}}$
2. if *v1*_*i*_ and *v2*_*i*_ are less than zero, $vc_{i}=-\sqrt{v1_{i}*v2_{i}}$
3. if the product (*v1*_*i*_ * *v2*_*i*_) is less than zero, $vc_{i}=+\sqrt{v1_{i}*v2_{i}}*\sqrt{-1}$, i.e., an imaginary number.

By extending the definition of geometric mean to the square root of a negative number as an imaginary one, CD ΔF(common) captures not only average up- or down-regulated genes through the real “vc” values, but also changes that occur in opposite directions on the two base diets through imaginary “vc” values.

A corresponding CD ΔS(common) was generated from CD ΔS(-F) and CD ΔS(+F).

### Error estimate for CD vectors

To estimate the error in the “v” value of each gene in the primary CD vectors, 100 null vectors were calculated from random pairs of mean-corrected expression data of individual animals, rather than the mean difference between groups. The mean-corrected expression sets from experimental and control animals were combined for this purpose, providing for 8 individual expression data and 7! possible pairs. The mean value of the 100 null vectors was close to zero. The procedure for calculating null vectors follows Clark et al. [17], except that sorting is omitted. It uses the same co-variance matrix and normalization factor as for the primary CD vectors. The standard deviations (SD) of the null vectors for each TranscriptClusterID was used as estimate for the error of “v_*i*_” values of the corresponding vectors.

For a secondary vector, such as CD ΔF(common), the SD of its “vc” values is derived from the two primary vectors as follows:

$$SDcommon_{i}=\sqrt{SD1_{i}*SD2_{i}}$$ (3)

### Cosine similarity calculations

Similarity of 2 vectors of identical dimensions is easily assessed by calculating the cosine of the angle between them: an angle of 0^o^ with a cosine value of 1 indicates perfect alignment while 90^o^ or cosine 0 reflects orthogonal vectors with complete dissimilarity.

Given two vectors, *A* and *B*, the cosine similarity, *cos(θ)*, is represented using a dot product A•B and magnitude ‖A‖ and ‖B‖ as:

$$\cos(\theta )=\sum_{j=1}^{n}Aj*Bj/(\sqrt{\sum_{j=1}^{n}Aj^{2}}*\sqrt{\sum_{j=1}^{n}Bj^{2}})$$ (4)

where Aj and Bj are components of vector A and B respectively [23]. The calculations were implemented in R [22]. Gene vectors subjected to analysis were sorted in the same gene space according to the criteria indicated in the legends.

### Extraction of top differentially expressed genes from CD vectors

The CD (R-package GeoDE, version 1) uses an empirical cut-off to select the top differentially expressed genes without taking into account possible errors of “v” values. This cut-off identified about ~7 k genes out of a total of ~30 k. To focus on the important genes, we developed 2 inclusion/exclusion criteria based on rank and error considerations:

1) We define an enrichment score (*ES*) for the gene’s “*v*” value and a cut-off based on the “*v*” value of a vector with equal contributions of all genes. Only genes with *ES* values greater than zero are included. In mathematical terms, for any gene with positional rank ‘*i* ‘, an enrichment score *ES*_*i*_ is defined as

$$ES_{i}=|v_{i}{}^{2}|-(1/n)$$ (5)

whereby *v*_*i*_ represents the DE value of each gene. *ES*_*i*_ = 0 when |*v*_*i*_^2^| = (1/*n*). (1/*n*) is the squared expression value for a vector where all genes contribute equally to DE, i.e., $\pm \sqrt{1/n}$. Excluded genes are those with *ES* values ≤ 0. To find all genes with (1/*n*), gene vectors are resorted in descending order of *ES* values and re-indexed in terms of positional rank ‘i*‘*. Then a cumulative distribution function (CDF) of [*v*_*i*_^2^ − (1/*n*)] is constructed, i.e.,

$$CDF(ES_{i})=\sum_{1}^{i}\left[v_{i}{}^{2}-(1/n)\right]$$ (6)

At the maximum of CDF(*ES*), i.e., at CDF(*ES*)_max_, *ES*_*i*_ ≃ 0, thus identifying a cut-off rank “*i*_*max*_”. Given the asymmetrical distribution of *ES*_*i*_, for all higher “*i*”s *ES*_*i*_ ≤ 0.

2) To balance rank of the “*v*” value for inclusion with the magnitude of the error for exclusion, the squared value of the estimated error for each gene’s “*v*” value is added to the CDF. The new function with error CE_*i*_ is defined as follows:

$$CE_{i}=CDF(ES_{i})+2*SD_{i}{}^{2}$$ (7)

If CE_*i*_ > CDF(*ES*)_max_, then the uncertainty of the “*v*” value is considered too high and the gene is excluded from the set of top most differentially expressed genes. This use of a CDF is based on a modification of a function used by Clark et al. [17] and implemented in Excel.

The Eqs 5–7 apply to both primary and secondary vectors and thus apply to both “*v*” and “*vc*”. Note, that there is change in the definition of ES_*i*_ between Eq 5 and subsequent ones in that only Eq 5 contains the absolute value of “*v*^*2*^”. This is necessary to appropriately rank imaginary “*vc*” values.

### Principal component analysis (PCA)

For PCA analysis, the expression data set for 200 TranscriptClusterIDs was analyzed using the prcomp() function in R [22]. The top most ranked TranscriptClusterIDs from the real part of CD ΔF(common) and a similarly calculated CD ΔS(common) were identified and their expression data were subjected to PCA analysis. The data matrix from all 16 animal samples was centered and variance normalized.

### Analysis by univariate methods

We also analyzed DE by two univariate methods: 1) ANOVA using Affymetrix Transcriptome Analysis Console (TAC) (Version 3.0); and 2) Bayesian Analysis of Variance for Microarrays [20] (BAMarray, http://www.bamarray.com) run at the highest stringency. ANOVA and BAM generate lists of individual genes whose expression has reached statistical significance. Statistical significance depends on baseline variance, fructose-induced fold-changes in expression, and prior knowledge of variance patterns. ANOVA generates a list of genes with associated mean expression changes and p-values, while BAM generates a list of genes with its own associated Zcut statistics. Fructose-induced genes were considered those on a single diet with p<0.05 with ANOVA and an absolute value of Zcut>2.4 with BAMarray, while fructose-specific genes were identified by these statistics on both diets.

### Quantitative polymerase chain reaction (qPCR)

qPCR was carried with a Qiagen custom 96-well RT^2^ PCR assay according to the supplier’s instructions with an ABI StepOnePlus thermocycler. The Qiagen validated primers were for Pck1 (Rn.104376), G6pc (Rn. 10992), Tmem50a (Rn. 957), Actb (Rn. 94978), Gapdh (Rn. 91450), Lrp2 (Rn. 26430), Agtrap (Rn. 8471), Hmgcs2 (Rn. 29594), and Slc13a2 (Rn. 10821). The plates contained controls for rat genomic DNA contamination, positive PCR, and reverse transcription. The raw signals were routinely monitored for amplification in the log-linear phase with the program LinRegPCR.exe [24] (http://www.hartfaalcentrum.nl) and analyzed by the Cy0 method which calculates initial rates of amplification based on the entire time course of amplification [25] (https://www.cy0method.org). The Cy0 method was implemented on a Mathcad15 program. The Cy0 method was shown to be superior to the Δ method in a large scale series of experiments [26]. Data were normalized with respect to the expression of Gapdh or Actb.

### Gene Ontology (GO) enrichment analysis

The enrichment analysis of the fructose-specific CD gene list was carried out with the Database for Annotation, Visualization and Integrated Discovery (DAVID, version 6.8, https://david.ncifcrf.gov). Questionable gene annotations were cross-checked with Genecards (https://www.genecards.org/). Erroneous entries, when realized during the analysis, were corrected (S7 Table) and parameters of enrichment, significance and false discovery rate (FDR) recalculated.

### Statistical methods

p < 0.05 was used as significance criterion for t-tests, ANOVA, and the EASE test used by DAVID (a modified Fisher exact test). BAMarray comes with its own statistics of “Zcut”. Absolute Zcut values of > 2.4 were considered statistically significant as indicated by the software program.

## Results

### Analysis of fructose-induced DE by the Characteristic Direction method

To test our hypothesis, and overcome the problem of interactions between a fructose supplement and the base diet, we studied the effects of fructose on two base diets, designated here as normal- and high-salt diet (Fig 1A). Expression data were generated from total RNA of superficial kidney cortex by the *Affymetrix RaGene-2_st* chip. The primary ProbetSetID expression data were summarized at the gene (TranscriptClusterID) level and analyzed for differential expression as shown in Fig 1B. With the CD method (Fig 1B, upper arrows), primary fructose-induced gene vectors with associated DE values were generated, designated as CD ΔF(NS) and CD ΔF(HS) for normal- and high-salt base diets, respectively. The normalized changes in expression values of the genes comprising these vectors ranged from about ±0.3 for the highest up-regulated and down-regulated genes to ±10^−9^ for the lowest ones. For comparison, a vector where all genes make the same contribution to overall differential expression, would have values of $\pm \sqrt{1/n}$, where *n* = number of genes, or ~±0.006 for our data set with ~30k genes. The apparent paradox of getting DE values lower than $\sqrt{1/n}$ in real data vectors is a consequence of the normalization so that when some DE values in a vector are > $\left|\sqrt{1/n}\right|$ others become < $\left|\sqrt{1/n}\right|$.

Useful criteria for a cut-off of differentially expressed genes have not been developed. For example, current implementation of CD (R-package GeoDE, version 1) uses an empirical cut-off based on an *in-silico* model. With this, we identified ~7,000 genes affected by fructose out of a total of ~30,000. This high fraction of identified genes did not provide useful insight into the physiological processes affected by fructose, the primary purpose of our study.

**Fig 2:**
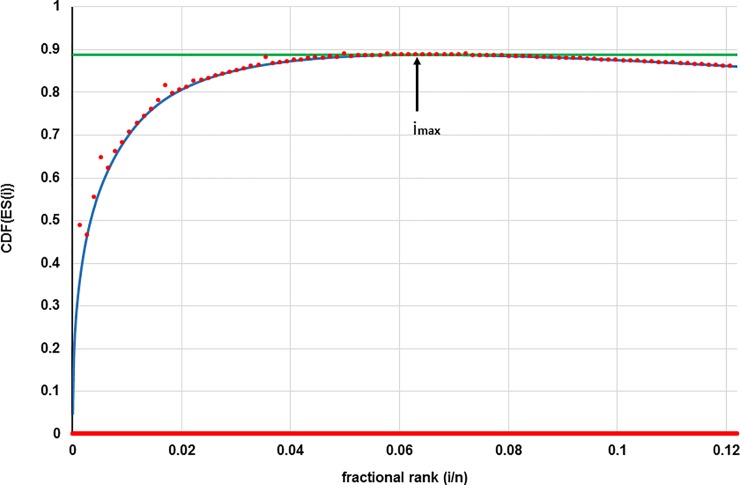
Selection of significant DE genes from CD ΔF(NS). Genes are selected based on their contribution to overall DE provided their variance is not too high. Each gene with DE value (*v*_*i*_) is assigned an enrichment score *ES*_*i*_ defined as *ES*_*i*_ = |*v*_*i*_^2^| − (1/*n*). To assess a gene’s contribution, genes are sorted in descending order of *ES*_*i*_ and then assigned a positional rank “*i*”, ranging from 1 to *n*, the total number of genes. The “blue line” represents the early portion of a plot of the cumulative distribution function $CDF(ES_{i})=\sum_{1}^{i}\left[v_{i}{}^{2}-(1/n)\right]$ (Eq 6 in Methods), versus “fractional rank” (*i*/*n*). This type of plot allows identification of the maximum value of the CDF (green line) and a fractional rank of (*i*_*max*_). At (*i*_*max*_), the gene’s |*v*_*i*_^2^| ≃ (1/*n*), the value expected for a vector where all genes make equal contributions. All genes with higher positional ranks than “*i*_*max*_” can thus be excluded on the basis that their |*v*_*i*_^2^| is less than (1/*n*) (or *ES*_*i*_ ≤ 0) and their contribution to overall DE is minor. To account for the uncertainty of the DEvalue the standard deviation (SD) is included. i.e., 2*SD^2^ of each gene’s DE value is added to its CDF(*ES*_*i*_) value (“red dots”) (Eq 7 in Methods). For illustration purposes, only every 40^th^ point is shown. The genes with red dots below the green line have a sufficiently high DE value and sufficiently low SD to be included in the significant DE genes in terms of induced fructose effects.

To address this problem, we developed a new algorithm to define the cut-off point for significant changes in expression called “rank/deviation”. As the name implies “rank/deviation” takes into account both a gene’s DE value and its standard deviation as demonstrated for CD ΔF(NS) in Fig 2 and explained in detail in the legend. With this new approach the number of significant genes differentially expressed by dietary fructose was 1814 and 1745 on normal- and high-salt diet, respectively (Table 1, S1 and S2 Tables). These fructose-induced gene sets contain many genes which are present on only one of the lists or whose expression changes go in opposite directions on the two diets. In fact, <10% of the differentially expressed genes on each diet are common and change in the same direction. This result indicates that the basal metabolic state determined by the type of base diet influences the effect of fructose supplementation on gene expression.

**Table 1:** Fructose diet-dependent differentially expressed genes of kidney cortex.

		differentially expressed genes/TranscriptClusterIDs		
Method	Experimental Condition	down	up	total
**Characteristic Direction (CD)**	± fructose–normal salt chow = CD ΔF(NS)	741	1073	1814
(for cut-off see Methods)	± fructose–high salt chow = CD ΔFHS)	1023	722	1745
	CD ΔF(signature)	56	83	139
	annotated CD ΔF(signature)	37	60	97
	Genes representing 80% of information from annotated CD ΔF(signature)	9	25	34
	common to CD, ANOVA, and BAMarray	1	5	6
**ANOVA (p < 0.05)**	± fructose–normal salt chow = ANOVA ΔF(NS)	830	756	1586
	± fructose–high salt chow = ANOVA ΔF(HS)	1044	1378	2422
	ANOVA ΔF(common)	51	57	108
	annotated ANOVA ΔF(common)	48	39	87
	common to ANOVA and BAMarray	14	8	22
	common to CD and ANOVA	3	7	10
**BAMarray (super accuracy)**	± fructose–normal salt chow = BAM ΔF(NS)	329	451	780
	± fructose–high salt chow = BAM ΔF(HS)	515	598	1113
	BAM ΔF(common)	17	15	32
	annotated BAM ΔF(common)	15	9	24
	common to CD and BAMarray	1	6	7

The low percentage of the significant fructose-induced genes common to both base diets prompted us to combine ΔF(NS) and ΔF(HS) by gene-wise calculation of the geometric mean of the DE values into a fructose-specific gene vector CD ΔF(common) (see Methods). Application of the cut-off algorithm to CD ΔF(common) yields a vector of 240 significantly changed genes (S1 Fig). Not all these genes change in the same direction on the two base diets. The 58% that change in the same direction constitute a fructose-specific CD ΔF(signature). This vector is comprised of 139 genes, of which 97 are annotated (Tables 1 and 2 and S3 Table). The remaining 42% with opposing fructose-induced changes indicate a substantial interaction of fructose with base diet.

**Table 2:** Partial list of annotated ΔF(signature) genes (complete list in S3 Table).

Rank	Gene	DE Value of fructose-specific, annotated genes	CDF (DE value)^2^ (%) of annotated CD ΔF(signature)	Annotation
1	Pck1 *+&	0.441	19%	Cytosolic Phosphoenolpyruvate Carboxykinase
2	G6pc *#&	0.341	31%	Glucose-6-phosphatase catalytic subunit
3	Gatm	0.248	37%	Mitochondrial L-Arginine-Glycine Amidinotransferase
4	Bhmt	0.175	40%	Betaine—Homocysteine S-Methyltransferase
5	Acsm3	0.173	43%	Mitochondrial Middle-Chain Acyl-CoA Synthetase
6	Slc25a10	0.168	46%	Mitochondrial Succinate Transporter
7	Miox	0.157	49%	Myo-Inositol Oxygenase
8	Aadat	0.155	51%	Mitochondrial Transaminase with broad substrate specificity
9	Klk1	0.151	53%	Glandular Kallikrein 1
10	Slc5a8	0.149	55%	Sodium-Coupled Monocarboxylate Transporter 1
17	Tkfc #	0.106	66%	Bifunctional ATP-Dependent Dihydroxyacetone Kinase/FAD-AMP
21	Slc2a5 +#	0.097	70%	Glucose/Fructose Transporter
22	Slc5a10 #	0.097	71%	Sodium-Glucose/Fructose Cotransporter 5, Sglt5
26	Khk +#	0.087	74%	Ketohexokinase of fructose to fructose-1-phosphate.
28	Hmgcs2 &	0.085	76%	Mitochondrial 3-Hydroxy-3-Methylglutaryl-CoA Synthase (ketogenesis)
44	Prss8 *	0.069	85%	Serine Protease 8 (Prostasin)
57	Slc13a2 *	0.058	90%	Renal Sodium-Dependent Dicarboxylate Transporter
93	Agtrap *	0.044	99%	Angiotensin II Receptor Associated Protein
94	Tmem50a *	0.044	99%	Transmembrane Protein 50A

* = genes identified as significant with all 3 analysis methods

+ = genes in GO terms "response to fructose" or "cellular response to fructose stimulus"

# = genes for enzymes/transporters of fructose metabolism

& = genes identified as significant in high-salt diet by qPCR using Actb as reference and 2-sided t-test.

**Fig 3:**
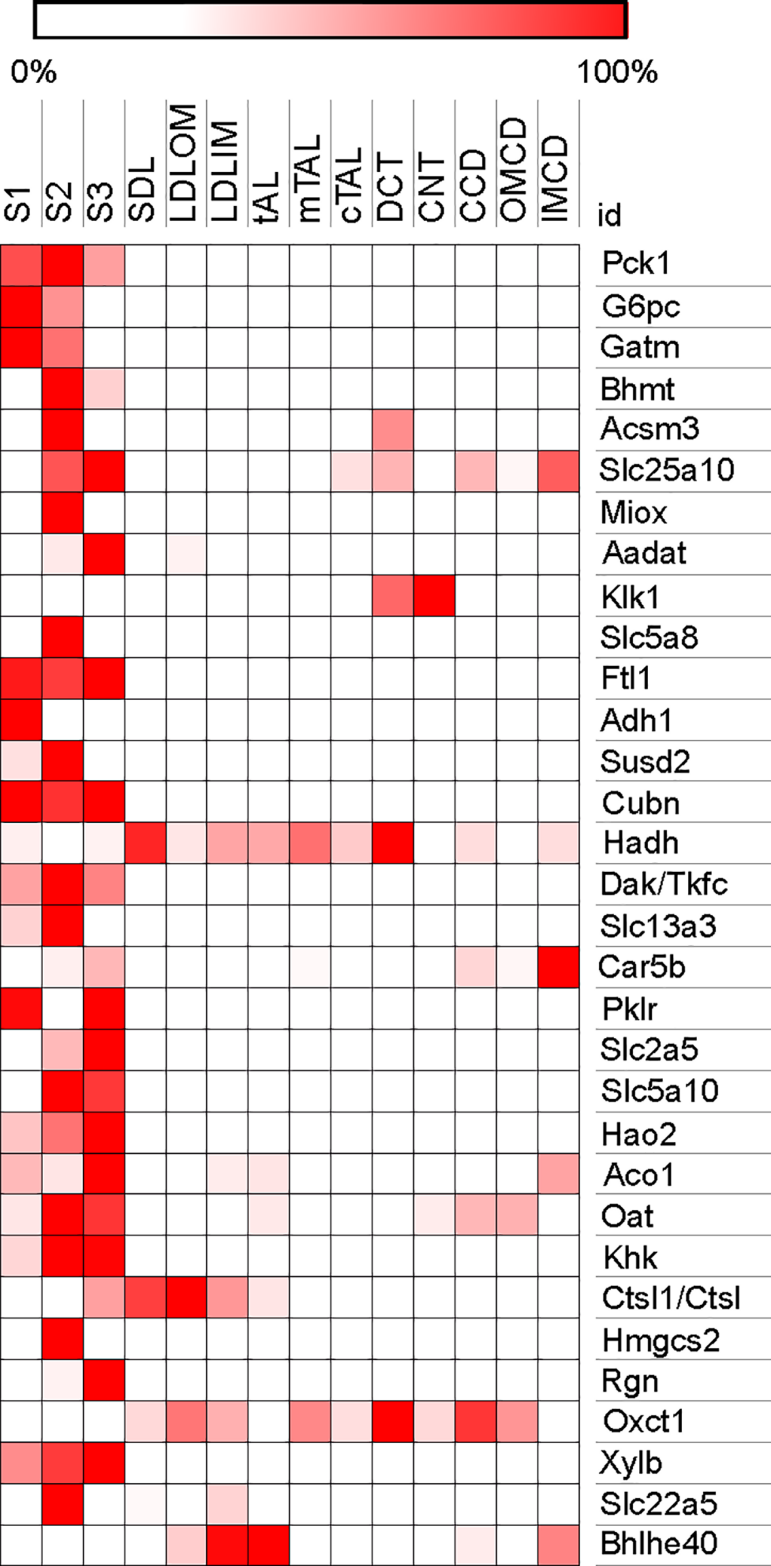
Distribution of expression of 32 of the top annotated fructose signature genes along the nephron. Expression data are from the database at https://hpcwebapps.cit.nih.gov/ESBL/Database/NephronRNAseq/All_transcripts.html [8]. Nephron segments: S1, S2, S3 = segment 1, 2, and 3 of proximal tubule, respectively; SDL = short descending limb of the loop of Henle; LDLOM and LDLIM = long descending limb of the loop of Henle in the outer and inner medulla, respectively; mTAL and cTAL = medullary and cortical segments of thick ascending limb, respectively; DCT = distal convoluted tubule; CNT = connecting tubule; CCD = cortical collecting duct; OMCD and IMCD = outer and inner medullary collecting duct, respectively. Attempts were made to map the top 34 genes of the fructose signature; whose DE represent 80% of the fructose signature DE. However, 2 genes are not present in the data base or have zero expression. The heat map was generated with Morpheus software https://software.broadinstitute.org/morpheus.

For the normalized CD vectors, the square of a gene’s DE value can be regarded as its fractional contribution to overall differential expression. Analysis of the annotated fructose signature in this respect reveals that the top 34 ranked genes account for 80% of the differential expression in this set. Thus, it is likely that the subset of 34 genes represents the primary response to fructose (Table 2). As shown in Fig 3, expression of 26 of these genes is predominantly or exclusively localized to the proximal tubule. The majority of the genes code for metabolic enzymes or transporters for metabolites as opposed to receptors, transcription factors, structural proteins, or secreted or degradative enzymes. The functions associated with these genes indicate a clear shift in the metabolic characteristics of the proximal nephron.

### Principal component analysis (PCA)

**Fig 4:**
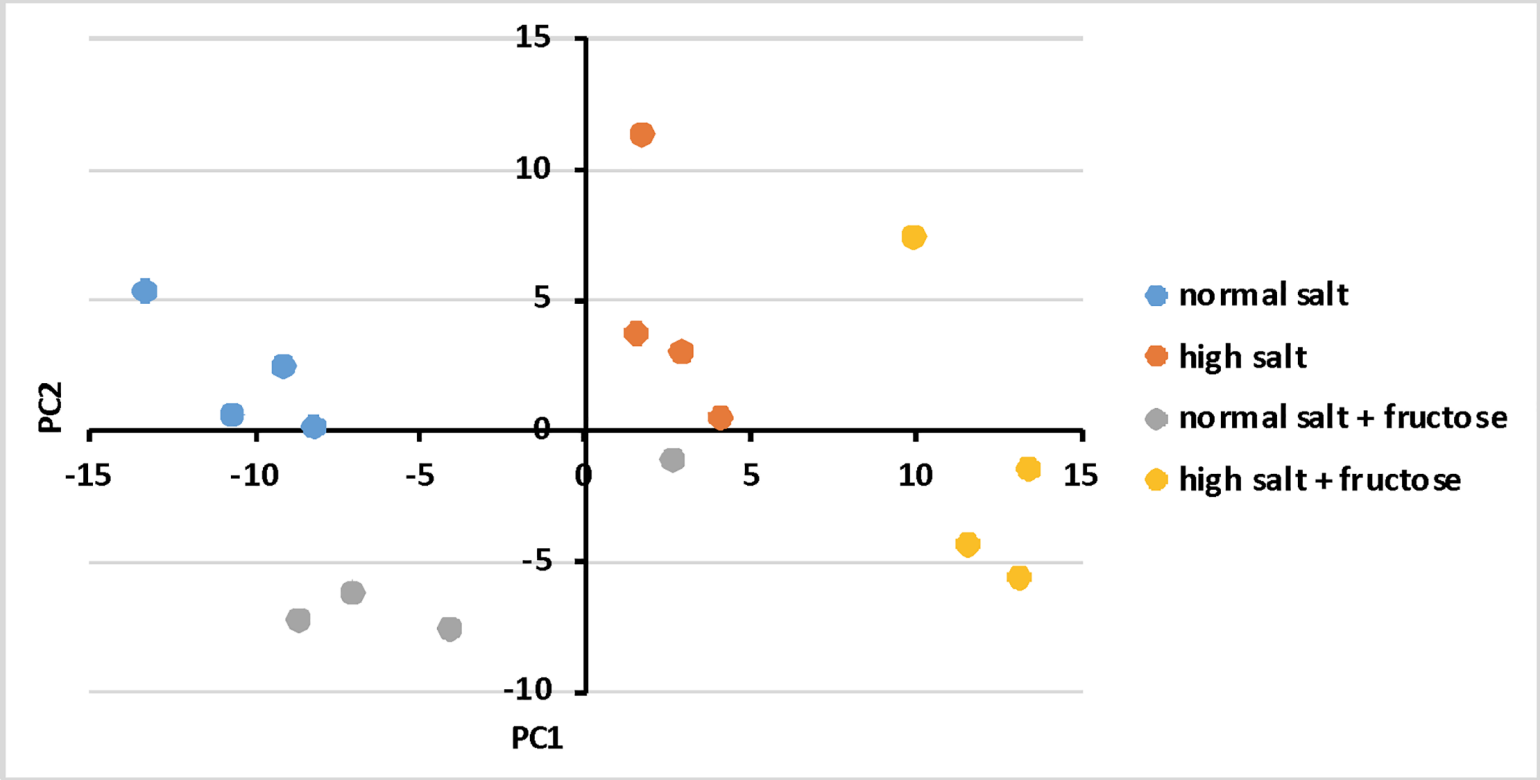
Principal component analysis (PCA) to distinguish among the 4 groups of animal treatments (±fructose on 2 different base diets). PCA analysis was carried out with the expression data of the 100 top fructose-specific plus 100 top diet-specific Affymetrix TranscriptClusterIDs, i.e., genes regardless of whether they are annotated or not.

PCA analysis [27] was used to study whether the CD selected genes could differentiate among different dietary treatments. PCA analysis was carried out with the expression data of the top 200 genes identified from CD ΔF(common) and CD ΔS(common), generated in analogous manner. Fig 4 shows how the expression data from each animal project onto the first two principle components. The four different dietary treatment groups can clearly be distinguished as they occupy different areas on the plot. These data also demonstrate that fructose alone and salt alone have different effects on gene expression, and that there is a significant interaction between these two parameters.

### Cosine similarity and contribution to total DE

**Fig 5:**
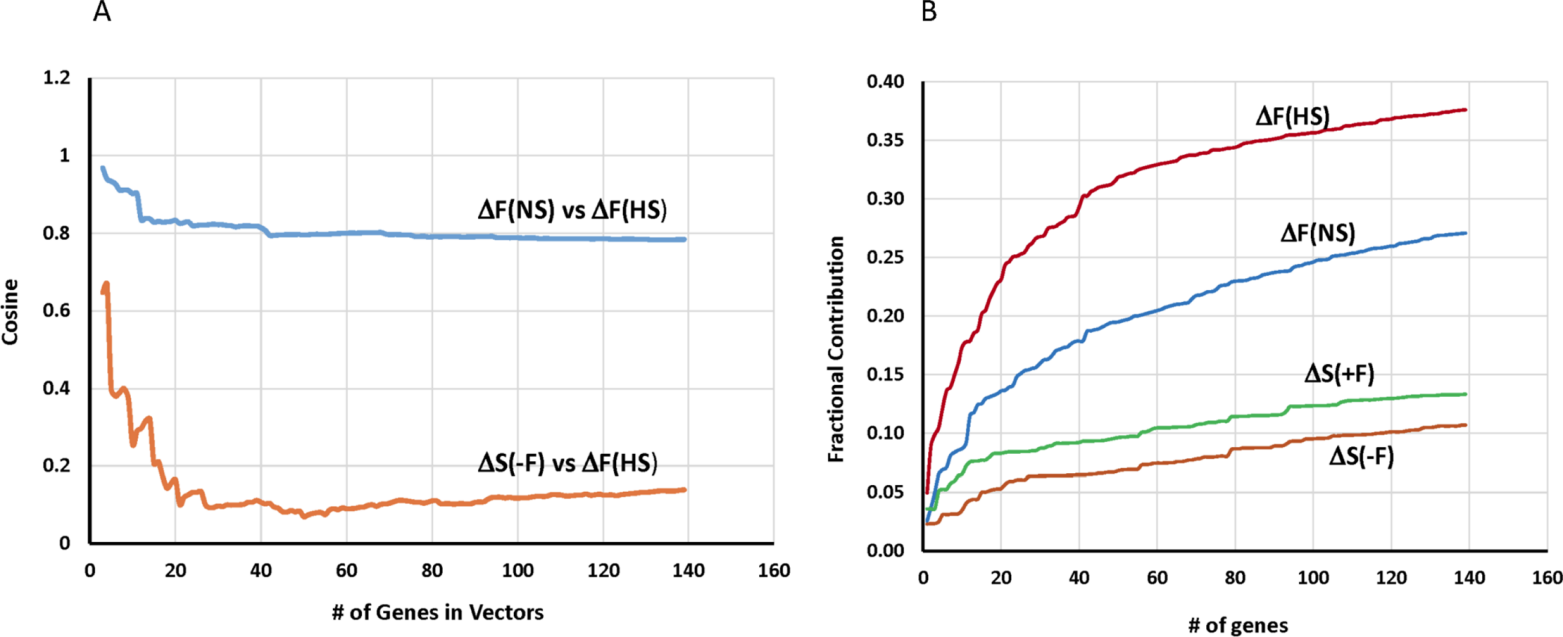
Similarity and importance of the fructose signature in CD ΔF(NS), ΔF(HS), ΔF(-F) and ΔF(+F). A) To compare similarity, vectors of just the fructose signature genes were extracted from CD ΔF(NS), ΔF(HS), ΔS(-F), and ΔS(+F) and the cosine of the angle between pairs of truncated vectors calculated. Truncation retains the top most genes sorted according to ΔF(signature) with the number of genes in the truncated vectors as indicated on the abscissa. A minimal, meaningful number of genes for comparison is 3, yielding the cosine of the angle between 2 vectors in 3D-space. The upper curve shows ΔF(NS) versus ΔF(HS) and the lower one ΔS(-F) versus ΔF(HS). B) Contributions of fructose signature genes to total differential expression in the different CD vectors. The fractional contribution to differential expression is calculated as the sum of the squared DE values (or (vector length)^2^) of the truncated CD vectors, sorted and truncated as in A.

The fructose signature is a vector of genes and their quantitative relationships in terms of induced expression. Thus, we next checked whether the fructose signature is well represented in the fructose-induced CD vectors ΔF(NS) and ΔF(HS) by measures of cosine similarity and contribution to total differential expression. For this purpose, CD ΔF(NS) and CD ΔF(HS) vectors were sorted according to the ranking of CD ΔF(signature) and the cosine similarity calculated between truncated CD ΔF(NS) and CD ΔF(HS) vectors (Fig 5A upper curve, blue). Indeed, the similarity was very high with a cosine value of 0.97 for the top 3 genes of the fructose signature and remained > 0.78 when all 139 fructose signature genes were included. In contrast, the behavior is very different when unrelated CD vectors are compared. For this purpose, the expression data were analyzed by CD for the effect of diet (high versus normal salt) and two gene vectors generated termed CD ΔS(-F) and CD ΔS(+F) in absence and presence of fructose, respectively (Fig 1A and Methods). Comparison between CD ΔS(-F) (effect of high-salt diet in the absence of fructose) and CD ΔF(HS) (effect of fructose with a high-salt diet), sorted also according to CD ΔF(signature), demonstrated dissimilarity with the exception of a single gene (Pck1) (Fig 5A, lower curve; orange). Pck1 is highly ranked in all 4 primary vectors (DE values of 0.22, 0.16, 0.19, and 0.16 for ΔF(HS), ΔF(NS), ΔF(+F), and ΔS(-F), respectively). Comparison of other combinations of vectors yielded cosine similarities in between the 2 curves of ΔF(NS) vs ΔF(HS) and ΔS(-F) vs ΔF(HS).

To assess significance of these cosine values, cosine similarity was calculated between ΔF(NS) and 100 random null vectors generated as described in the Methods section. These cosine values ranged from -0.25 to +0.25 with a peak around 0, as expected for null vectors (S2 Fig). Thus, the observed high cosine values between 0.97 and 0.78 (Fig 5A) for the signature genes in ΔF(NS) vs. ΔF(HS) comparison indicate significant similarities in the response of gene programs to fructose on both base diets.

Another parameter of interest is the extent of the contribution of the fructose signature genes to total differential expression, based on the squared DE value. Fig 5B shows cumulative distribution functions of fractional contributions as a function of fractional gene rank for all 4 CD vectors sorted in same descending order as the signature genes. For the fructose-induced vectors ΔF(HS) and ΔF(NS), the contributions of the 139 signature genes account for 38% and 27%, respectively, of total DE. Interestingly, the signature genes are also represented in the base diet-induced vectors ΔS(-F) and ΔS(+F) with 11% and 13%, respectively. This means that the high-salt diet affects many of the same genes as fructose, but obviously not necessarily in the same direction or magnitude as judged from the cosine similarity of ΔS(-F) vs ΔF(HS) in Fig 5A.

### Comparison of CD with univariate models and qPCR

We next compared the fructose effects found by the CD method to those from two widely used univariate models, ANOVA and BAMarray, the software implementation of BAM [20] (Fig 1B, lower arrows). Table 1 provides a comparison of the number of fructose-induced genes con a single diet and fructose-specific genes common to both base diets identified by all 3 analysis methods as well as the ones common to different methods, with p ≤0.05 for ANOVA and |Zcut| > 2.4 for BAMarray. The order of magnitude of identified genes on a single diet was similar with all 3 methods, but provides little insight because < 10% are common to normal- and high-salt diets in any of the methods. This is a well recognized problem, as illlustrated by the fact that with ANOVA none of the fructose-induced genes on a single diet reached significance after adjusting for multiple comparisons.

Only six fructose-specific genes were identified by all three analysis methods and are mapped onto the CD ΔF(signature) in Table 2 (marked by “*”). Interestingly, 2 of these are the most highly ranked genes (Pck1 and G6pc), while the other 4 are ranked below 40. Thus, many highly ranked genes of CD ΔF(signature) in between were not identified by the univariate methods.

qPCR is widely used in conventional transcriptome analyses by univariate methods to confirm the differential expression of identified genes. Therefore, expression by qPCR was determined for five of the common significant genes, Hmgcs2, and reference/control genes (Gapdh, Actb, Lpr2). The selection was made to check both very high ranking (Pck1, G6pc with ranks 1 and 2) and very low ranking (Slc13a2, Agtrap, Tmem50a with ranks 57,93, and 94) common significant genes as well as one identified only by CD with intermediate ranking (Hmgcs2 with rank 28). Overall, the precision of qPCR in identifying differentially expressed genes was only significant when Actb was used as a reference but not Gapdh (S5 Table) and revealed as a positive correlation between microarray and qPCR data (S3A and S3B Fig). The qPCR data showed statistical significance for Hmgcs2 for both base diets and for Pck1 and G6pc with the high-salt diet.

### Gene Ontology (GO) enrichment

To assess consistency of the results with prior knowledge, the annotated genes of CD ΔF(signature) and the fructose-specific univariate gene lists were analyzed with the recently updated DAVID 6.8 bioinformatic resource [28] (https://david.ncifcrf.gov/) in terms of enrichment for GO terms from “Biological Process” and mitochondrial “Cellular Component” (Table 3). The mitochondrial terms were included because mitochondrial metabolism plays a key part in changes of cellular metabolism and we previously reported enhanced respiration in response to angiotensin II in fructose-fed rats [11]. The terms “response to nutrient”, “cellular response to fructose stimulus”, and “response to fructose” were significantly enriched (genes marked by “+” in Table 2). Furthermore, 11 of the 13 significantly enriched processes are concerned with responses to nutrients or metabolism.

**Table 3:** Enrichment of GO terms for CD ΔF(signature) from DAVID (v. 6.8).

GO:biological process	Identified Genes	Fold Enrichment	EASE score (modified Fisher exact p-value)	Benjamini p-value	FDR
**0007584~response to nutrient**	**CUBN, GATM, HMGCS2, OXCT1, PKLR, KLK1**	**23**	**4.60E-06**	**0.001**	**6.09E-05**
**0046835~carbohydrate phosphorylation**	**KHK, TKFC, XYLB**	**66**	**0.001**	**0.115**	**0.011**
**0032868~response to insulin**	**KHK, HMGCS2, HADH, PCK1**	**20**	**0.001**	**0.087**	**0.013**
**0071422~succinate transmembrane transport**	**SLC25A10, SLC13A3**	**354**	**0.005**	**0.320**	**0.070**
**0015744~succinate transport**	**SLC25A10, SLC13A3**	**354**	**0.005**	**0.320**	**0.070**
**0055085~transmembrane transport**	**SLC5A8, SLC13A3, SLC22A5, SLC5A10**	**10**	**0.007**	**0.342**	**0.094**
**0071332~cellular response to fructose stimulus**	**SLC2A5, PCK1**	**213**	**0.009**	**0.349**	**0.114**
**0014823~response to activity**	**OXCT1, HADH, PCK1**	**17**	**0.012**	**0.390**	**0.150**
**0009749~response to glucose**	**KHK, CTSL, PKLR**	**15**	**0.016**	**0.433**	**0.192**
**0032869~cellular response to insulin stimulus**	**HMGCS2, PKLR, PCK1**	**13**	**0.021**	**0.487**	**0.246**
**0009750~response to fructose**	**KHK, SLC2A5**	**89**	**0.022**	**0.461**	**0.252**
**0001889~liver development**	**HMGCS2, ACO1, RGN**	**11**	**0.029**	**0.525**	**0.319**
**0006880~intracellular sequestering of iron ion**	**FTL1L1, FTL1**	**59**	**0.032**	**0.538**	**0.353**
**GO:cellular constituents (relevant to metabolism)**					
**0005739~mitochondrion #**	**Gatm, Acsm3, Slc25a10, Adh1, Hadh, Car5b, Hmgcs2, Aadat, Oat, Oxct1**	**3.5**	**0.001**	**3.75E-04**	**0.014**

# corrected for incorrect inclusion/exclusion of genes in GO term, fold-enrichment, p-values etc

A similar analysis for the gene lists of univariate ANOVA or BAM yields less support for fructose-induced metabolic processes. For example, GO:terms of “response to nutrients” or “response to fructose” are not enriched (see S6 Table for GO:Biological Process and S7 Table for GO:Cellular Component:mitochondrion).

## Discussion

We report here for the first time a transcriptome signature induced by dietary fructose in a mammalian kidney. This CD ΔF(signature) consists of a vector of 139 genes characterized by a quantitative relationship of DE values among them. Several different physiological and methodological aspects of the research approach and analysis protocols are important for success in this project: 1) metabolism of fructose by the proximal renal nephron; 2) design considerations for dietary experiments; and 3) greater power of the multivariate CD method to extract DE genes in spite of high baseline variance as compared to univariate analysis methods and to generate quantitative DE gene vectors; and. 4) a new cut-off algorithm called rank/deviation to select significant genes with the CD method.

Experimental validation of the results is based on consistency with prior knowledge of metabolism, in particular renal one, and internal consistency based on several tests discussed below in detail.

### Effect of dietary fructose on proximal tubule metabolism

Expression changes were measured in superficial renal cortex samples on the assumption that the data reflect effects in the proximal tubule. Superficial cortex as proxy is justified because proximal tubules comprise most of the mass, and fructose metabolizing enzymes are present only in this segment [7–9]. It has the advantage of rapid sample processing, thereby avoiding in-vitro, post-mortem changes of mRNA. Furthermore, the bulk of the most important identified DE genes were proximal tubule-specific (Fig 3) indicating that the fructose-induced genomic effects occur indeed mainly in the proximal tubule under our experimental conditions. While some of the genes, such as mitochondrial Slc25a10, Prss8, or Agtrap are expressed in multiple segments, differential expression may only represent proximal nephron effects, primarily because that is where fructose metabolism occurs.

Metabolic studies have provided considerable knowledge about changes under conditions of food abundance and starvation as well as in response to a fructose diet at times of food abundance, although much more for liver than proximal tubule. In terms of overall body metabolism, the liver and proximal tubule share many features as they both provide fuel for other tissues depending on nutritional state and type of diet. For example, they share the capacity for gluconeogenesis, lipogenesis, ketogenesis, and fructose metabolism via fructose-1-phosphate [29, 30]. The knowledge that proximal tubules metabolize fructose via fructose-1-phosphorylation has been used previously to link fructose-dependent renal damage to local fructose metabolism; inhibition or deletion of the enzyme ketohexokinase, which catalyzes fructose-1-phosphorylation, was shown to protect animals and humans from renal damage caused by fructose [31–34].

Consistency of our results with prior knowledge was evaluated through Gene Ontology enrichment (DAVID) of biological processes and mitochondrial association, and literature on metabolism. Of the 95 annotated genes recognized by DAVID (2 Affymetrix annotated genes were not recognized) 24 were enriched in processes related to metabolism in general or specifically to fructose (Table 3). The 24 genes were in the top 34 of the annotated fructose signature. This is a remarkable enrichment given that the database for DAVID has been derived predominantly from non-proximal tubule tissues, and reflects mainly knowledge derived from metabolism in liver and other non-renal tissues.

Gene enrichment analysis is informative for processes where overall rates depend on contributions from many steps, but not for those characterized by rate limiting enzymes. Interestingly, the 2 top ranked DE genes are Pck1 and G6pc, which encode the cytosolic enzymes phospho-enolpyruvate carboxykinase and glucose-6-phosphatase, respectively, are key enzymes that catalyze irreversible steps in glycero- and gluconeogenesis [35]. Glyceroneogenesis, in turn, is key to de-novo triglyceride synthesis [36, 37], i.e., lipogenesis. Other genes in the ΔF(signature) have also been associated with lipogenesis (Slc15a10 and Aco1 [38–40]). Thus, the most important metabolic processes influenced by fructose appear to be gluconeogenesis and lipogenesis. This conclusion is in line with the known metabolic capacities of the proximal tubule.

A change of metabolism towards gluconeogenesis and lipogenesis is further supported by the many mitochondrial genes whose expression changes (S7 Table). Mitochondrial tricarboxylic acid metabolism plays a particular role in glycero- and gluconeogenesis from amino acids. Under conditions of excess amino acid degradation relative to oxidative energy needs, excess tricarboxylic acid cycle metabolites are transported into the cytosol for conversion to glycerol, glucose, and fatty acids. This process has been termed cataplerosis [41–43] and has been investigated at the flux, but not the transcriptome level. Fructose-induced cataplerosis is suggested by the increased levels of Gatm and Slc25a10, coding for mitochondrial transamidinase and dicarboxylic acid transporter, respectively. Excess mitochondrial fuel is further suggested by increases in Hmgcs2 which codes for a mitochondrial enzyme considered rate limiting for ketogenesis [44].

A remarkable cluster of five genes in ΔF(signature) with elevated expression is concerned with fructose transport and metabolism (marked by “#” in Table 2). An increase in ketohexokinase by dietary fructose had been noted before in liver [45]. The ability of fructose to increase expression of genes concerned with its own transport and metabolism would suggest the possibility of accelerating rate increases with time on a fructose diet.

Together these results indicate that 20% fructose in the drinking water for 7 days is sufficient to initiate genetic reprogramming of renal metabolism with increased gluconeogenesis, ketogenesis—and lipogenesis, i.e., important physiological processes known to occur in proximal tubules. In addition, they indicate genetic programs for cataplerosis and fructose absorption and metabolism. While fructose-induced metabolism and starvation share the processes of renal gluconeogenesis and ketogenesis, a distinguishing feature between them is lipogenesis with fructose and typically lipolysis in starvation [46–48].

### Design considerations for dietary experiments

One of the known difficulties in studying the effect of dietary manipulations is that observed changes may depend on the base diet and base metabolic state used as control [16]. To circumvent this issue, we studied differential expression caused by fructose in the drinking water with two different base diets (Fig 1). With the CD method of analysis, this 2 x 2 experimental design results in 2 different fructose-induced vectors, namely ΔF(NS) and ΔF(HS) from which the high ranking DE genes with similar effects on both diets could be extracted as ΔF(signature).

By design we chose to study the effects of an amount of fructose as may be caused by seasonal fruit consumption, such as bears gorging on blueberries. With consumption of fructose for only 7 days, the animals do not have confounding factors of longer-term fructose consumption, namely elevated plasma glucose, insulin resistance, or metabolic syndrome [5, 11]. This absence is important because of our interest in the primary gene program in response to fructose and the likelihood that confounding factors would change gene expression and thus lead to erroneous functional assignments. A number of rodent models have been established to generate metabolic syndrome or insulin resistance with fructose diets. These models use up to 60% of their caloric intake in the form of fructose and time frames from 3 to greater than 12 weeks[1]. Transcriptome signatures obtained under such conditions would most likely include the pathology rather than the evolutionary, adaptive genetic response to fructose. Given the absence of metabolic syndrome in our rat model, the ΔF(signature) generated in this study most likely represents the primary and important higher order genes whose expression is altered by renal fructose metabolism.

It is noteworthy that CD ΔF(HS) reflects DE under conditions of blood pressure levels that are about 15 mm Hg higher than for CD ΔF(NS). Blood pressure is thought to be an important physiological parameter for proximal tubule function. Thus, CD ΔF(HS) likely contains differentially expressed genes that represent responses to blood pressure.

### Development of the rank/deviation cutoff for CD gene vectors

Currently the CD package [17] comes with an empirical cut-off algorithm based on an in-silico model that has little rational basis in terms of physiological function or variance of affected genes. Case in point, this published method identified ~7,000 fructose-affected genes, about 25% of the total 30,000. Such a high fraction provides little useful information in terms of true physiological responses. To address this problem, we devised a new algorithm to define the cut-off point. Our method weights: 1) the contribution of genes to overall DE, and 2) the variance in the DE values. The contribution of genes to overall DE is quantified as the absolute value of the square of the DE value, and only genes are considered significant that make greater contributions than those expected for a vector in which all genes make equal contributions. The cut-off is easily identified by ranking genes according to their contribution (Fig 2). The variance was estimated from the distribution of DE values of null vectors from random pairs of mean-corrected expression data from each set of experimental and control animals. With this new approach the fructose-induced gene sets represent about 6% of the total (Table 1) and likely contain not only primary, but also secondary and higher response genes. Less than10% of the fructose-induced genes on each diet are common and change in the same direction. The rank/deviation method is particular effective in extracting the most important DE genes that respond to fructose in a similar manner on both base diets (S1 Fig) and constitute CD ΔF(signature).

### Multivariate CD method versus univariate ones

The CD method takes into account the information inherent in the covariance matrix of the expression data, i.e., co-regulation, in addition to the usual mean difference between experimental and control data, and generates a DE vector that quantitatively reflects the relative contribution of each gene to overall differential expression. This multivariate approach is well suited to extract information about gene programs and is appropriate when the major source of gene expression variance is in transcriptional regulation of many genes together rather than fluctuations in individual genes.

The quantitative nature of these vectors has several advantages because well-developed mathematical tools are available for averaging and comparing similarity of different vectors or even sub-vectors corresponding to different gene programs and thereby extracting new information. We took advantage of these vector properties in calculating average fructose-induced effects on the base diets in the form of ΔF(common). Averaging needs to be accomplished using the geometric, rather than the arithmetic, mean of the values in ΔF(NS) and ΔF(HS) because these are normalized vectors. ΔF(commons) actually contains imaginary numbers for genes whose fructose-induced expression changes in opposite direction on the 2 different base diets.

Another advantage is the ability to assess similarity of the response to a perturbation under different conditions or with different animal models. To assess whether fructose induced similar gene programs on normal- and high-salt diets we calculated the cosine of the angle between vectors of the fructose signature genes from CD ΔF(NS) and CD ΔF(HS) (Fig 5A). This angle does not depend on the magnitude of the response, but only the relative response of fructose signature genes to each other. To evaluate the significance of the observed value of >0.78, it was compared to the cosine values between ΔF(NS) and 100 random CD null vectors that are not only dissimilar to ΔF(NS), but also contain in their ensemble the noise information of the expression data. These latter values had a distribution around 0 with a range of ±0.25 (S2 Fig) so that the conclusion of similar fructose responses of animals on both base diets is justified. Therefore, it is likely that the fructose signature genes are affected by fructose under a wide range of base diets, not just the 2 actually tested.

One explanation for the greater power of the multivariate CD method in comparison to conventional univariate methods is that identification of DE genes is less dependent on the magnitude of the variance. To assess whether indeed the CD method will pick up differentially expressed genes despite relatively high baseline variance, we compared the coefficients of variation, i.e., the normalized SD, of the microarray data between two sets of genes from CD ΔF(signature): 1) several fructose transporters and metabolizing enzymes identified only by CD ΔF(signature) (marked by “#” in Table 2) and 2) two low ranked genes (Agtrap and Tmem50a), ranked 93 and 94 out of a total of 97 of the annotated CD ΔF(signature), but also identified as significant by univariate methods (marked by “*” in Table 2). The expectation was that univariate methods identify genes based on the ratio of fold-change-to-variance, i.e., a low baseline variance is required to enable recognition of small fold-changes in expression. Indeed, while the two low ranked genes have low fold-changes, they also have very low coefficients of variation, which enabled their identification by ANOVA and BAM, in addition to CD. In contrast, the higher ranked fructose metabolizing enzymes have higher fold-changes, but also higher coefficients of variation so that they were not identified by the univariate methods (S4 Table). Renal proximal tubule metabolic processes are known to respond to changes of nutritional and acid/base status, which in turn depend on other variables, such as circadian rhythm and microbiome, so that high variance is not unexpected for expression of genes coding for metabolic enzymes. Thus, the importance of low variance for identifying DE genes by univariate analysis methods can explain their lack of power compared to the CD method. Certain crucial assumptions underlying univariate methods of analysis have been previously criticized as inappropriate for expression data of complex multicellular organisms[49]. Interestingly, qPCR has the same drawbacks in terms of requiring low biological variance so that our qPCR analysis of several genes turned out to be no more informative than the microarray data (S5 Table).

The greater power of the CD method over univariate analysis was clearly evident in the gene enrichment analysis by DAVID. Only the CD method identified sufficient numbers of genes relevant for metabolism so that the biological processes of “response to nutrients” or “response to fructose” from the Gene Ontology data base were significantly enriched (S6 Table).

Apart from providing physiological context information on annotated genes, the CD method also brings out the gap in knowledge between molecular biology and function. 29% of the TranscriptClusterIDs of CD ΔF(signature) are not annotated and their functions are unknown at present. Some of these genes without known function are highly ranked and would be expected to deserve high priority in future research. For example, TranscriptClusterIDs 17809429 and 17826846 (RGD1560687) are ranked #4 and #5 below Pck1, G6pc, and Gatm (S3 Table).

## Conclusion

The specific fructose-induced gene signature reported here constitutes a new type of physiologic metric for characterizing physiological phenomena. Our analysis shows that the CD method allows extraction of gene programs, i.e., co-regulated genes, from differential expression experiments. With more than one base diet, it is possible to extract information that is valid over a wider range of base diet composition. The expression changes of the fructose signature genes are consistent with fructose-induced programs of gluconeogenesis, lipogenesis, ketogenesis, and cataplerosis in proximal tubules. Thus, these genes provide a solid starting point for more targeted research into detailed downstream effects of fructose metabolism to sort out the players responsible for the pathology associated with high fructose. While renal fructose metabolism is clearly implicated in mediating pathogenesis of acute renal injury [31–34], importance of various downstream events mediating the cellular/metabolic reprogramming remains controversial [50–52]. The approach of combining analysis by CD with averaging of gene vectors and the newly described “rank/deviation” cutoff method has wider importance beyond fructose and the proximal tubule. First, it has greater power than univariate methods; second, the quantitative nature of signature gene vectors allows rational integration of information from differential expression experiments with different types of dietary manipulations or with different animal or cell models, e.g., by similarity analysis. Therefore, this analysis method can substantially contribute to mapping transcriptome profiles to physiological functions. The strategy and methods are clearly applicable to many dietary problems.

## Supporting information

S1 Fig Selection of significant DE genes from CD ΔF(common). (PDF)

S2 Fig Frequency of cosine similarity between CD ΔF(NS) and 100 CD null(NS) vectors. (PDF)

S3 Fig Correlation between Affymetrix microarray and qPCR measurements. (PDF)

S1 Table Fructose-induced CD ΔF(NS) vector. (XLSX)

S2 Table Fructose-induced CD ΔF(HS) vector. (XLSX)

S3 Table Fructose-specific CD ΔF(signature). (XLSX)

S4 Table Analysis of coefficient of variation for highly ranked genes for fructose metabolizing enzymes. (XLSX)

S5 Table qPCR analysis of selected genes. (XLSX)

S6 Table Enrichment of GO:Biological processes for annotated ΔF(signature). (XLSX)

S7 Table Enrichment of GO:0005739~mitochondrion for annotated CD DF(signature) within GO:Cellular constituents. (XLSX)

S8 Table Weight of animals at sacrifice. (XLSX)
