# Supplementary material for: Transcriptome signature for dietary fructose-specific changes in rat renal cortex: A quantitative approach to physiological relevance
Source: PLoS One. 2018 Aug 1;13(8):e0201293. doi: 10.1371/journal.pone.0201293 (PMC6070266; doi:10.1371/journal.pone.0201293)

Transcriptome signature for dietary fructose-specific changes in rat renal cortex:

a quantitative approach to physiological relevance

Agustin Gonzalez-Vicente[1], Jeffrey L. Garvin[1], and Ulrich Hopfer[1]*

[1] Department of Physiology & Biophysics, Case Western Reserve University, Cleveland, OH

* Corresponding author

ulrich.hopfer@case.edu

Supporting Information

S1 Fig. Selection of significant DE genes from CD ΔF(common).

To assess the contribution of each gene's DE value to overall DE expression, the genes are sorted in descending order of the absolute value of their squared DE value and then assigned a positional rank "*i*", ranging from 1 to *n,* the total number of genes. Each gene is then assigned an enrichment score ES_i defined as $ES_i = |v_i^2| - (1/n)$. The "blue line" represents the early portion of a plot of the cumulative distribution function $CDF(ES_i) = \sum_1^i \left[v_i^2 - (1/n) \right]$ (equation 6 in Methods) versus "fractional rank" (*i*/*n*). This type of plot allows identification of the maximum value of the CDF (red line) and a fractional rank of (i_{max}). At (i_{max}), the gene's $|v_i^2| \simeq (1/n)$ the value expected for a "no-DE" vector. All genes with higher positional ranks than "i_{max}" can thus be excluded on the basis that their

$|v_i^2| \le (1/n)$ and their contribution to overall DE is minor. The “brown dots” represent the sum of each gene’s CDF value plus $2*SD^2$ of its DE value (equation 7 in Methods). Only genes with brown dots below the red line have a sufficiently high DE value and sufficiently low SD to be included in the high ranking DE genes in terms of fructose effects.

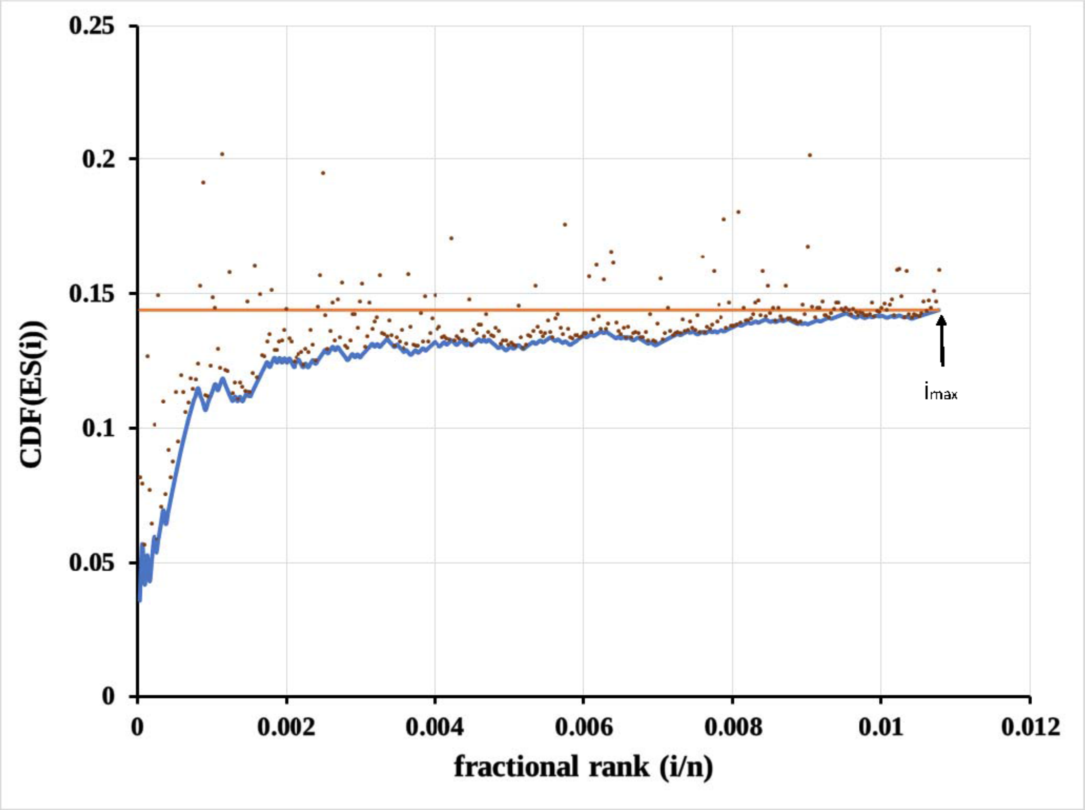

Supplement: S1 Fig — (PDF) [file pone.0201293.s001.pdf]
