# Supplementary material for: Transcriptome signature for dietary fructose-specific changes in rat renal cortex: A quantitative approach to physiological relevance
Source: PLoS One. 2018 Aug 1;13(8):e0201293. doi: 10.1371/journal.pone.0201293 (PMC6070266; doi:10.1371/journal.pone.0201293)
Supplement: S2 Fig — (PDF) [file pone.0201293.s002.pdf]

Transcriptome signature for dietary fructose-specific changes in rat renal cortex:

a quantitative approach to physiological relevance

Agustin Gonzalez-Vicente[1], Jeffrey L. Garvin[1], and Ulrich Hopfer[1]*

[1] Department of Physiology & Biophysics, Case Western Reserve University, Cleveland, OH

* Corresponding author

ulrich.hopfer@case.edu

Supporting Information

S2 Fig. Frequency of cosine similarity between CD ∆F(NS) and 100 CD null(NS) vectors.

Null vectors were generated from the complete expression data set from animals on the normal-salt diet after mean correcting separately the groups with and without fructose. Null vectors were calculated by CD from random pairs of data from individual animals, the co-variance of the entire data set, and the normalization factor for CD ∆F(NS) (see Methods: Error Estimate for CD vectors in Methods). Cosine values were calculated in R between truncated vectors comprising the 139 genes of the fructose signature of CD ∆F(NS) and 100 CD null ∆F(NS) vectors. The vectors were sorted so that the gene sequence was identical and responding to CD ΔF(NS). The frequency of binned results is plotted. Results are similar to those with full-length vectors.

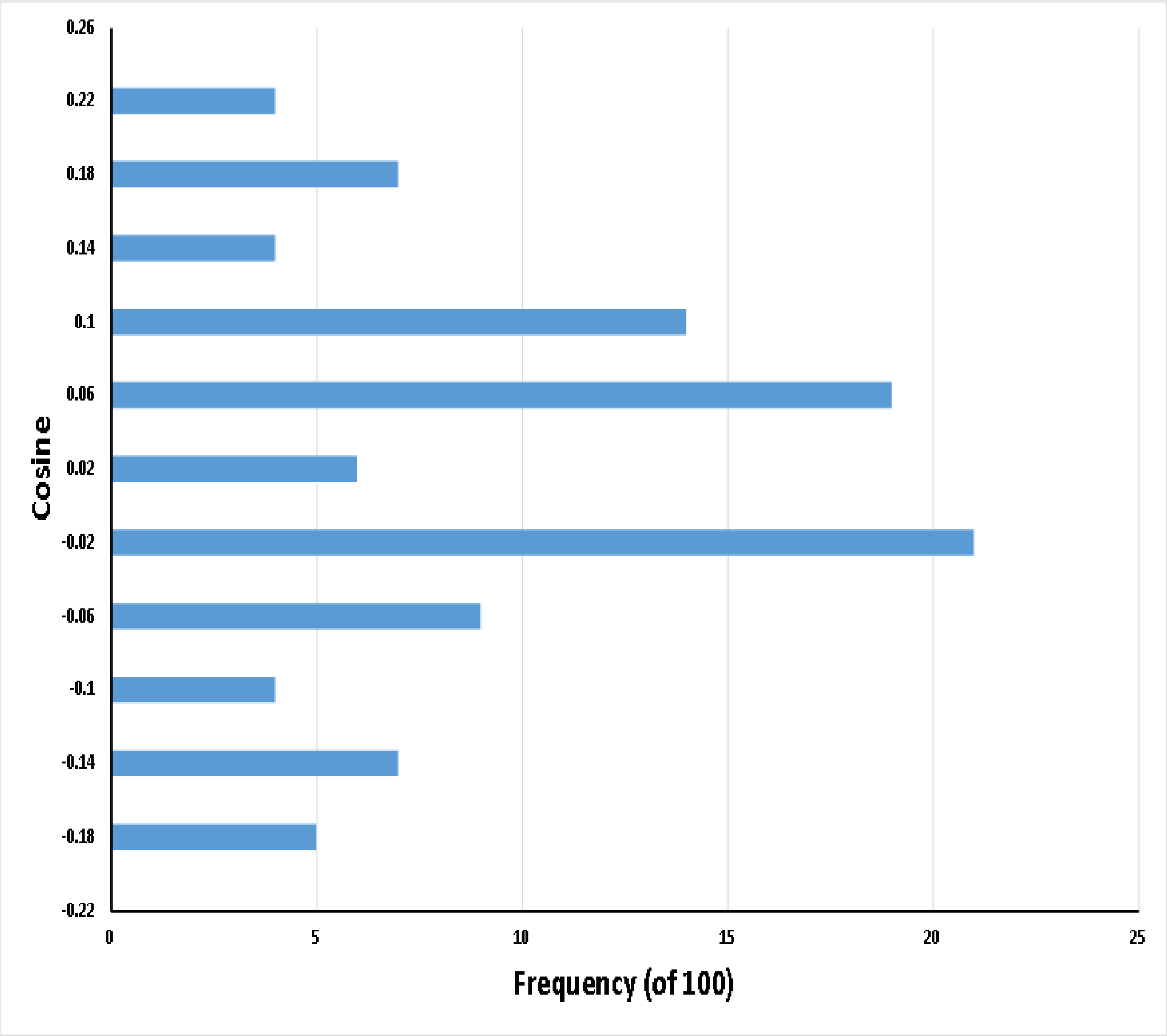

0.26
0.22
0.18
0.14
0.1
0.06
0.02
-0.02
-0.06
-0.1
-0.14
-0.18
-0.22
Cosine
0
5
10
15
20
25
Frequency (of 100)
