# Supplementary material for: Transcriptome signature for dietary fructose-specific changes in rat renal cortex: A quantitative approach to physiological relevance
Source: PLoS One. 2018 Aug 1;13(8):e0201293. doi: 10.1371/journal.pone.0201293 (PMC6070266; doi:10.1371/journal.pone.0201293)
Supplement: S3 Fig — (PDF) [file pone.0201293.s003.pdf]

Transcriptome signature for dietary fructose-specific changes in rat renal cortex: a quantitative approach to physiological relevance

Agustin Gonzalez-Vicente[1], Jeffrey L. Garvin[1], and Ulrich Hopfer[1]*

[1] Department of Physiology & Biophysics, Case Western Reserve University, Cleveland, OH

* Corresponding author

ulrich.hopfer@case.edu

Supporting Information

S3 Fig. Correlation between Affymetrix microarray and qPCR measurements.

Gene expression was corrected for Actb and have the same scale (log2). qPCR data are expressed as “-Cy0”. Error bars, when present, represent standard errors of the mean from the 4 animal replicates in each group. Absence of error bars indicate non-significance of correlation. A) Correlation between animals on normal-salt diet ±fructose. B) Correlation between animals on high-salt diet ±fructose.

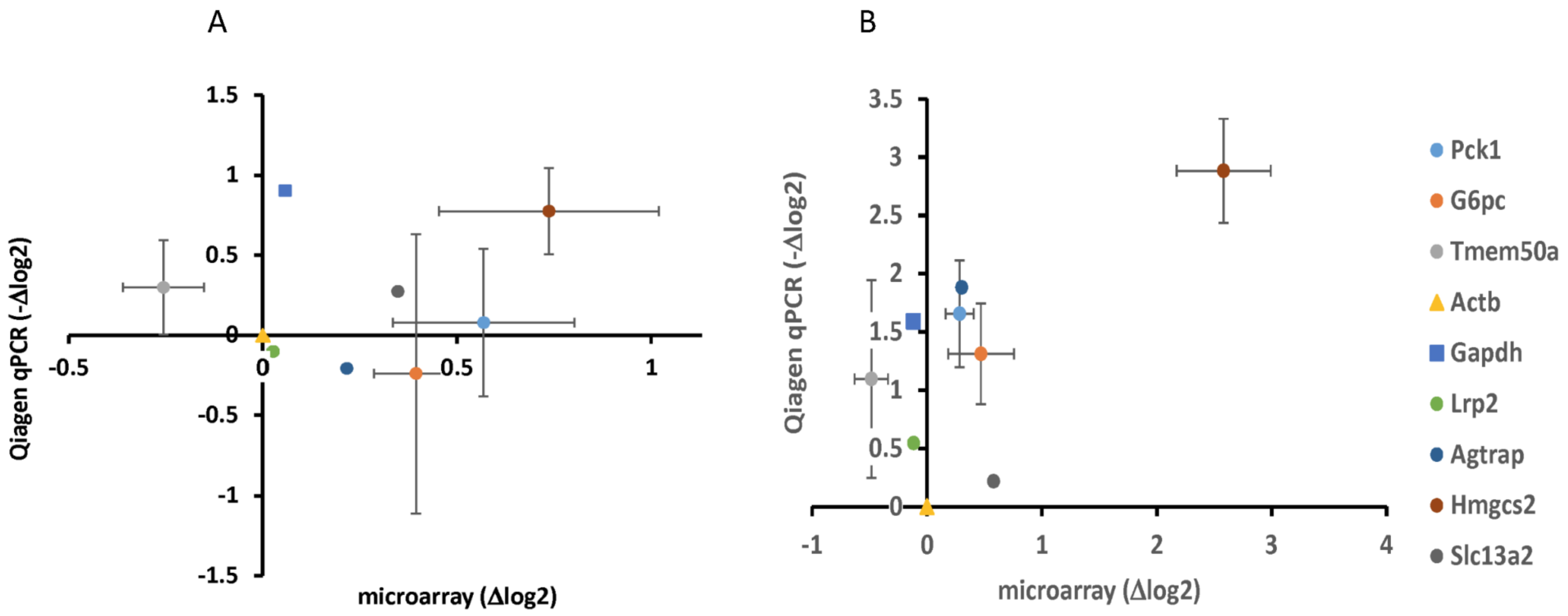

A
B
Qiagen qPCR (-Δlog2)
microarray (Δlog2)
1.5
1
0.5
0
-0.5
-1
-1.5
0.5
3.5
3
2.5
2
1.5
1
-1
0
1
2
3
4
Pck1
G6pc
Tmem50a
Actb
Gapdh
Lrp2
Agtrap
Hmgcs2
Slc13a2
